# Novel nonadride, heptadride and maleic acid metabolites from the byssochlamic acid producer *Byssochlamys fulva* IMI 40021 – an insight into the biosynthesis of maleidrides†
†Electronic supplementary information (ESI) available: Details of any supplementary information available should be included here. See DOI: 10.1039/c5cc06988b



**DOI:** 10.1039/c5cc06988b

**Published:** 2015-10-09

**Authors:** Agnieszka J. Szwalbe, Katherine Williams, Daniel E. O'Flynn, Andrew M. Bailey, Nicholas P. Mulholland, Jason L. Vincent, Christine L. Willis, Russell J. Cox, Thomas J. Simpson

**Affiliations:** a School of Chemistry , Bristol University , Cantock's Close , Bristol , BS8 1TS , UK; b School of Biological Sciences , University of Bristol , Bristol Life Sciences Building , 24 Tyndall Avenue , Bristol , BS8 1TQ , UK; c Syngenta , Jealott's Hill International Research Centre , Bracknell , Berkshire , RG42 6EY , UK; d Leibniz Universität Hannover , Institute of Organic Chemistry , Schneiderberg 1B , 30167 Hannover , Germany

## Abstract

The filamentous fungus *Byssochlamys fulva* strain IMI 40021 produces (+)-byssochlamic acid 1, its novel dihydroanalogue 2 and four related secondary metabolites. Agnestadrides A, 17 and B, 18 constitute a novel class of seven-membered ring, maleic anhydride-containing (hence termed heptadride) natural products. The putative maleic anhydride precursor 5 for both nonadride and heptadride biosynthesis was isolated as a fermentation product for the first time and its structure confirmed by synthesis. Acid 5 undergoes facile decarboxylation to anhydride 6. The generic term *maleidrides* is proposed to encompass biosynthetically-related compounds containing maleic anhydride moieties fused to an alicyclic ring, varying in size and substituents.

## Abstract 2

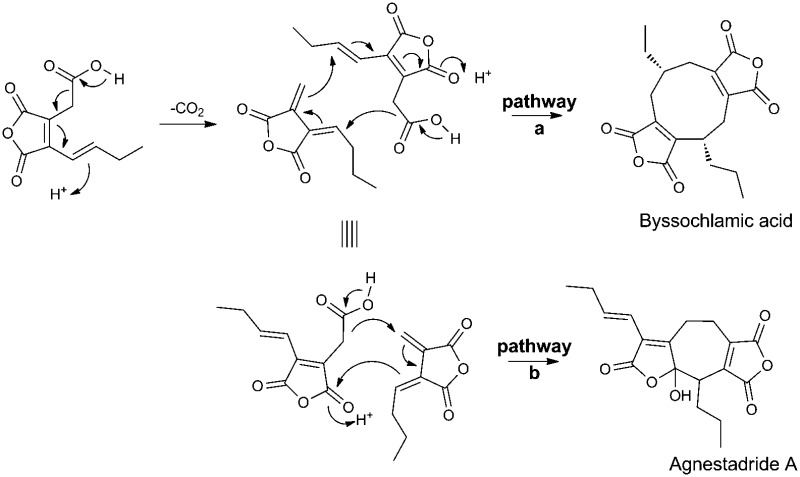
Byssochlamic acid and the novel heptadride, agnestadride A are biosynthesised *via* alternative dimerisations of a maleic acid monomer.

The collective name “nonadrides” was introduced by Barton and Sutherland for fungal metabolites containing a nine-membered ring fused to one or two maleic anhydride moieties in 1965.^1^ The first of these, glaucanic acid **3** and glauconic acid **4** were isolated from *Penicillium glaucum*,^2^ by Wijkman and reported in 1931. Soon afterwards the pseudo-symmetrical analogue, byssochlamic acid **1** was isolated from the common food contaminant *Byssochlamys fulva* by Raistrick and coworkers.^3^ However the structures of **1** and **4** were only established some 30 years later by X-ray analysis.^4,5^ Barton and coworkers subsequently reisolated **4** from *Penicillium purporogenum* and began considering the biosynthesis of the nonadrides.^6^ The *cis* relative stereochemistry of the ethyl and propyl groups of byssochlamic acid was confirmed by X-ray analysis of the bis*-p*-bromophenylhydrazide.^5^ The absolute configuration of **1** was established by classical degradative methods,^7^ and subsequently confirmed by enantiospecific synthesis of (+)-byssochlamic acid.^8^

All three metabolites, **1**, **3** and **4**, were hypothesised to be biosynthesised *via* alternative dimerisation modes of a C_9_/C_10_-maleic acid precursor **5** and/or **6**, and evidence for this was obtained by feeding studies with radiolabelled **6**.^9^ Many more nonadride metabolites have since been reported, *e.g.* heveadride **7**,^10^ an isomer of **1**, and others which are expected to be biosynthesised through dimerisation of differently substituted maleic anhydride units, including scytalidin **8**,^11^ and castaneolide **9**.^12^ More complex examples include rubratoxin A **10**, and its dihydro-analogue rubratoxin B **11**,^13^ and phomoidrides A **12** and B **13**.^14^ Cornexistin **14**, is a nonadride unique in having a single anhydride unit.^15^ Viburspiran **15** ^16^ and zopfiellin **16**,^17^ feature 8-membered carbocyclic rings and so have been classed as octadrides. Notably, all of the compounds belonging to this family reported so far have been isolated from fungi.
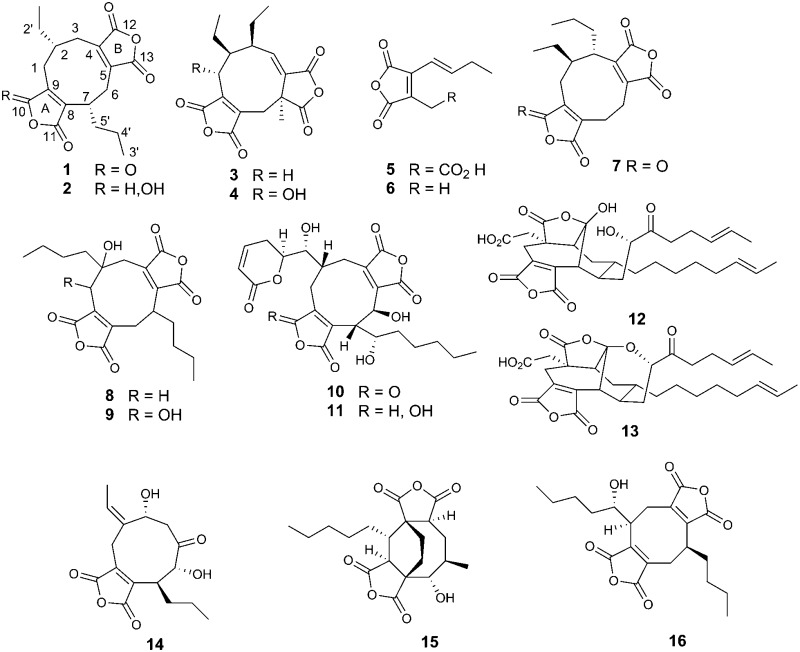


Pursuing a general interest in the biosynthesis of nonadrides and other related fungal compounds, we re-analysed the secondary metabolite profile of *B. fulva* to assess its nonadride-related metabolites in greater detail. This has led to isolation and characterisation of a new analogue, dihydro-byssochlamic acid **2**; two novel metabolites agnestadride A **17** and agnestadride B **18**, both containing 7 membered rings which we have therefore termed heptadrides; and as a natural product for the first time, the proposed nonadride precursor, maleic anhydride **5** along with its decarboxylated derivative **6**.

*B. fulva*, was grown as described by Raistrick^3^ in static culture in Czapek-Dox liquid medium with glucose as the sole carbon source for between 7 and 30 days. The crude ethyl acetate extracts were analysed by LCMS. Byssochlamic acid **1** (C_18_H_20_O_6_) was detectable after 7 days of static fermentation (Fig. 1, 15.4 min) and large amounts (>50 mg L^–1^) could be isolated after 4 weeks. Its identity was confirmed as (+)-byssochlamic acid by comparison of 1D ^1^H and ^13^C NMR spectra, and optical rotation comparison with literature values.^8^ Detailed analysis of 2D NMR spectra (COSY, HSQC and HMBC) allowed all signals in the ^1^H and ^13^C NMR spectra to be fully assigned for the first time (Table S1, ESI†).

**Fig. 1:**
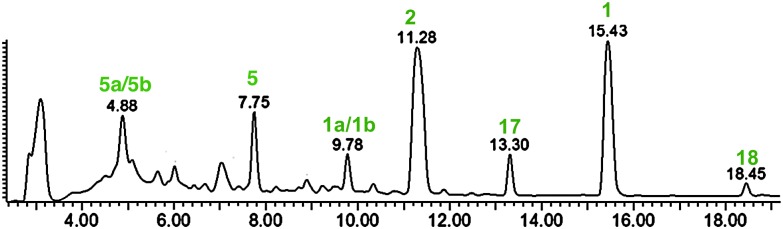
HPLC **(**DAD) chromatogram of *B. fulva* extract.

The second-most abundant component of the extract eluted at 11.3 min (Fig. 1). The new compound had a UV absorption characteristic for nonadrides (*λ*_max_ 211, 260 nm), ionised well in the negative ESIMS mode and had the same fragmentation pattern as byssochlamic acid **1** – the only difference being that all of the peaks appeared at *m*/*z* values two units higher. HRESIMS analysis confirmed the molecular formula to be C_18_H_22_O_6_, corresponding to a reduced form of **1**. Dihydrobyssochlamic acid **2** was purified by mass-directed preparative HPLC. The NMR spectra (Table S1, ESI†) were compared with those of byssochlamic acid **1**. A new peak at 95.9 ppm in the ^13^C NMR spectra correlated (HSQC) to H-10 at 5.71 ppm confirmed the presence of a hemi-acetal in **2**. Consistent with the loss of the carbonyl, the resonances of C-11 and C-9 appeared at higher chemical shifts (172.2 and 160.7 ppm respectively), and the C-8 resonance moved upfield to 130.6 ppm. Key HMBC signals included correlations between 1-CH_2_ protons (2.42/2.32 ppm) and C-8, C-9 and C-10; C-9, C-8 and C-11 but not C-10 correlated also to H-7 (2.81 ppm), consistent with the lactol hydroxyl being attached to C-10. Moreover, 6-CH_2_ (3.03/2.60 ppm) showed correlations with C-8, C-4, C-5 and C-13 (130.1, 139.1, 139.9 and 168.5 ppm respectively) but not C-11 or C-9 (171.8 and 160.3 ppm). Interestingly, closer inspection of the ^1^H NMR spectrum showed the presence of one major singlet at 5.71 ppm consistent with the hemi-acetal adopting one major configuration (68%) but three other singlet methine signals were apparent at 5.80 (16%), 5.84 (5%), and 5.93 (11%) ppm respectively. While one of these minor components is likely to be due to the epimeric lactol, the other two signals may be due to regioisomeric lactols. Other nonadride lactol analogues are known, *e.g.* rubratoxin B **11** ^13^ and dihydro- and tetrahydroepiheveadrides (*e.g.* **7** R = H, OH, * = *S*) have been isolated from *Wicklowia aquatica*, along with the corresponding lactones.^18^ In phomoidride A **12**, one of the anhydrides is found as a hemi-acetal due to a transannular C–C bond, and in phomoidride B **13** as a full acetal due to further linkage to a side chain hydroxyl.^14^

LCMS chromatograms of extracts from older cultures of *B. fulva* (*ca.* 30 days) showed a new peak at 13.3 min with the same molecular weight as byssochlamic acid **1** but a slightly different UV spectrum. It was isolated by HPLC (∼1 mg L^–1^ culture) and HRESIMS confirmed it to have the same molecular formula (C_18_H_20_O_6_) as byssochlamic acid. However, ^1^H NMR analysis (Table S2, ESI†) showed the presence of a 1-butenyl side chain. The HSQC spectrum revealed only nineteen hydrogens attached to carbon, suggesting that the other belonged to a hydroxyl. The presence of a further propyl substituent linked to an otherwise uncoupled CH, and two mutually coupled methylenes were obvious from the COSY spectrum (Fig. 2a). The characteristic signals for one maleic anhydride were apparent at 143.3 (C-11), 165.3 (C-12), 164.8 (C-13) and 144.9 (C-14) ppm. The remaining 4 carbons resonances at 168.5 (C-6), 127.0 (C-5), 153.4 (C-8) and 104.0 (C-7) ppm were indicative of an α,β-unsaturated ester/lactone and an acetal, consistent with the hydroxylactone moiety indicted in bold in Fig. 2a. Further detailed analysis of the HMBC data (Fig. 2b) was consistent with the seven-membered carbocycle, which we name agnestadride A **17**. The key correlations were seen from the butenyl H-4 (6.08 ppm) to C-5, C-6 and C-8, and from H-16 (3.54 ppm) to C-7, and 9-CH_2_ (3.01/2.66 ppm) to C-5, C-7 and C-8 to firmly locate the hydroxylactone ring. Similarly, correlations from 9-CH_2_ to C-10 (20.6 ppm) and C-11, and 10-CH_2_ (3.05/2.52 ppm) to C-11, C-12 and C-13 locate the maleic anhydride moiety.
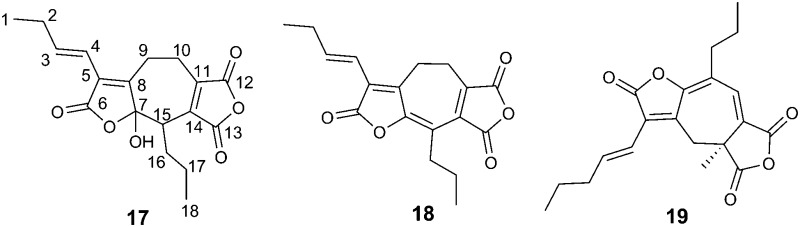


**Fig. 2:**
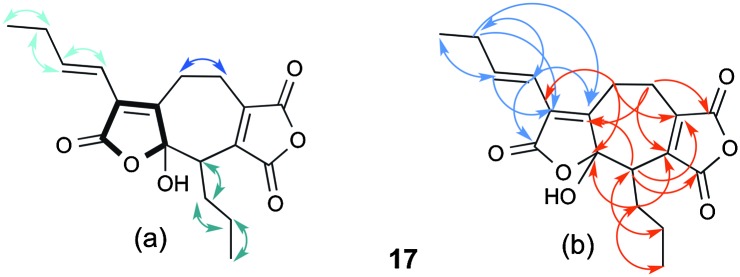
(a) COSY correlations of three isolated spin systems of **17**. (b) HMBC correlations.

A less polar new compound was detected (18.4 min in Fig. 1) and was isolated (1 mg L^–1^) using mass-directed preparative HPLC. It had a mass of 314 (peak of *m*/*z* 313 [M – H]^–^ in the negative ESIMS spectrum), corresponding to a dehydrated derivative of **17**. The formula was confirmed by HRESIMS analysis to be C_18_H_18_O_5_. In contrast with other colourless metabolites isolated from *B. fulva* compound **18** was bright yellow. The ^1^H NMR spectrum revealed that **18** also contained a pendant 1-butenyl side chain. A total of 18 protons were observed in the ^1^H NMR spectrum (Table S2, ESI†). There were three ^13^C NMR resonances (166.2, 164.6 and 163.1 ppm) characteristic for anhydride/unsaturated lactone carbonyls, and 6 quaternary olefinic carbons. The C-7 acetal and C-15 methine signals in 18 were absent, being replaced by two quaternary olefinic carbons. This data is fully consistent with compound **18**, named agnestadride B, being a dehydrated form of agnestadride A. The structure is supported by HMBC correlations, from H-4 to C-5 and C-6, as well as from 17-CH_2_ to C-15. To our knowledge, no heptadrides have been previously reported as natural products. Baldwin and co-workers, investigating biomimetic dimerisation of putative nonadride maleic anhydride monomers isolated a heptadride structure **19** which was also reported as being an intense yellow compound.^19^

Barton and coworkers suggested that maleic anhydride **6** is the precursor of **1**.^6^ Although indirect and direct evidence has accumulated for this hypothesis,^6,20,21^ anhydride **6** has never been observed as a component of *B. fulva* fermentations. Subsequent feeding studies on the phomoidrides utilising synthetic precursor analogues by Sulikowski and coworkers^22^ indicated that the monomeric nonadride precursor unit may be a carboxylated species, *e.g.* **5** for byssochlamic acid. In an attempt to find **5** or **6** in extracts of *B. fulva*, we searched extracted ion chromatograms of fresh extracts and identified a candidate peak at 7.7 min (Fig. 1). This metabolite formed ions of *m*/*z* 209.5, 165.5 in the negative ESIMS, which matched masses of ions expected for **5** ([M – H]^–^ and [M – H – CO_2_]^–^ respectively) and *m*/*z* 211.5, 193.3 in the positive ESIMS ([M + H]^+^ and [M + H – H_2_O]^+^ respectively). This compound was purified, but identification by full NMR (Table S3, ESI†) and HRESIMS analysis surprisingly showed it to be **6**, the decarboxylated form of **5**. Reinjection of **6** into the LCMS showed that it had a different retention time to **5**, suggesting that it had decarboxylated during purification and/or subsequent analysis. So to confirm the structure and study its stability maleic anhydride **5** was synthesised as shown in Scheme 1 based on previous work by Sulikowski.^22^ Commercially available mucobromic acid **20** was protected as silyl ether **21** in 79% yield. (*E*)-2-(But-1-en-1-yl)benzo[*d*][1,3,2]dioxaborole **22** prepared *via* the hydroboration of 1-butyne with catecholborane^23^ underwent Suzuki cross-coupling with **21** to give bromide **23** regio-selectively (as proven by HMBC). No bis-coupling was observed and the *trans*-geometry of the alkene was confirmed by a 16 Hz coupling constant in the ^1^H NMR spectrum. Palladium-catalysed coupling of the bromide **23** with silyl enol ether **24** (prepared separately by treatment of *tert*-butyl acetate with LDA and TBSCl)^24^ afforded ester **25** in an excellent yield of 93%. Removal of the TBS group with HF–pyridine was sluggish, giving yields of **26** typically between 30–46%. The resulting lactol **26** was then oxidised efficiently by Dess–Martin periodinane to **27** and final ester hydrolysis with TFA generated anhydride **5** in 66% yield.

**Scheme 1:**
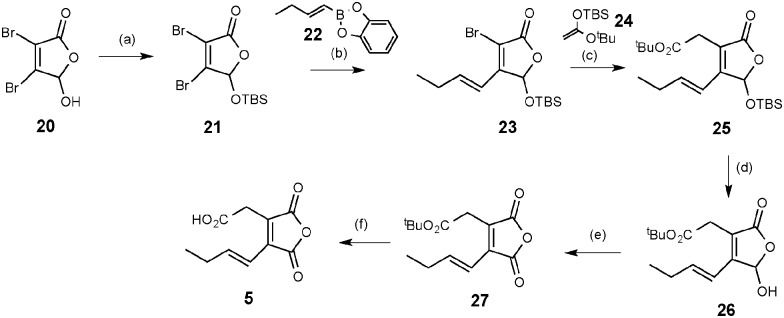
Synthesis of anhydride **5**. *Reagents and conditions*; (a) TBSCl (1.2 eq.), imidazole (1.5 eq.), DMAP (0.05 eq.), DCM : DMF (6 : 1), r.t., 22 h, 79%; (b) (*E*)-2-(but-1-en-1-yl)benzo[*d*][1,3,2]dioxaborole **22** (1.2 eq.), Pd(PPh_3_)_2_Cl_2_ (0.05 eq.), KOAc (2 eq.), PhMe, H_2_O, reflux, 16 h 79%; (c) 1-(*tert*-butyldimethylsilyloxy)-1-*tert*-butoxyethylene (2.04 eq.), Pd(PTol_3_)_2_Cl_2_ (0.05 eq.), KOAc (2 eq.), THF, reflux, 16 h, 93%; (d) HF–Py (70 : 30) (3 eq.), THF, 0 °C to r.t., 18 h, 46%; (e) DMP (3 eq.), DCM, 0 °C to r.t., 16 h, 86%; (f) TFA (excess), 66%.

The identity of naturally occurring **5** was confirmed by comparing its LCMS characteristics with the data collected for the synthetic product. The synthetic compound was observed to undergo decomposition to **6** spontaneously and decomposition was complete in under 48 h. The less polar peak at 20.6 min (Fig. S2, ESI†) had a similar UV spectrum to **5**, however it did not ionize well (the ESI spectrum shows a *m*/*z* 165 with a *m*/*z* of 209 but only in very concentrated samples), which was consistent with the loss of the ionisable carboxylate group. In addition the chemical shifts of peaks present in the ^1^H NMR spectrum of the impure naturally occurring **5** matched those of the synthetic analogue (Table S3, ESI†). Interestingly anhydride **6**, did not show any signal by ELS (Evaporative Light Scattering) detection and attempts to purify it by HPLC resulted in yields much lower than anticipated. This was attributed to volatility of **5**, and was confirmed by HPLC of a sample that had been allowed to evaporate at room temperature. On re-addition of solvent HPLC analysis showed that *ca.* 96% of the compound had evaporated.

Byssochlamic acid **1** exists in equilibrium with its mono-hydrated diacid forms which appear together at 9.8 min in the LCMS chromatogram (**1a**/**1b** in Fig. 1), as indicated by the characteristic [M + H_2_O – H]^–^ ion (*m*/*z* 349.2) in the negative ESIMS spectrum. This peak appeared in chromatograms of purified byssochlamic acid **1** and of a purified mixture of **1a**/**1b**. When re-injected, this reforms an equilibrium mixture with byssochlamic acid **1**. ^1^H NMR spectra showed that the ring-closed form **1** is preferred in organic solvent, while the ring-open forms **1a**/**1b** are also present in aqueous solvents. Similar behaviour is observed for other maleic anhydride containing metabolites including compounds **5** (see peaks at 7.35 and 4.88 min^–1^ in Fig. 1) and **6** (Fig. S3, ESI†) and dihydrobyssochlamic acid **2**, and for related maleic anhydrides isolated from *P. variotii*.^25^

Previous work^26^ had provided evidence for biosynthesis of byssochlamic acid *via* dimerisation of maleic anhydride **6** the decarboxylated form of **5**. However, it would appear likely that **5** is the actual precursor, decarboxylation providing the *exo*-methylene intermediate **28** necessary for cyclisation with a second molecule of **5** as indicated in Scheme 2 (pathway a). Tautomerisation of the first formed macrocycle leads to byssochlamic acid **1**. The heptadrides, agnestadrides A and B can be accounted for by a different mode of dimerisation of these intermediates as indicated in Scheme 2 (pathway b). Formal Michael addition of the anion derived by decarboxylation of **5** now occurs on the other end of the diene system of **28**. In the *in vitro* studies^19^ leading to **19** strong base was required to trigger dimerisation. Although presented in Scheme 2 as synchronous processes, stepwise mechanisms are equally valid at this stage. Interestingly diene **28** has been isolated as waquafranone B, along with waquafranone A **29** and several epiheveadrides from *W. aquatica.* ^18^ Also, the *exo*-methylene compound **30** has been isolated, as tubingenoic acid, from *Aspergillus tubingensis*,^27^ along with the hexyl analogue of **5**, itself a metabolite of several *Aspergillus* species.^26,28,29^

**Scheme 2:**
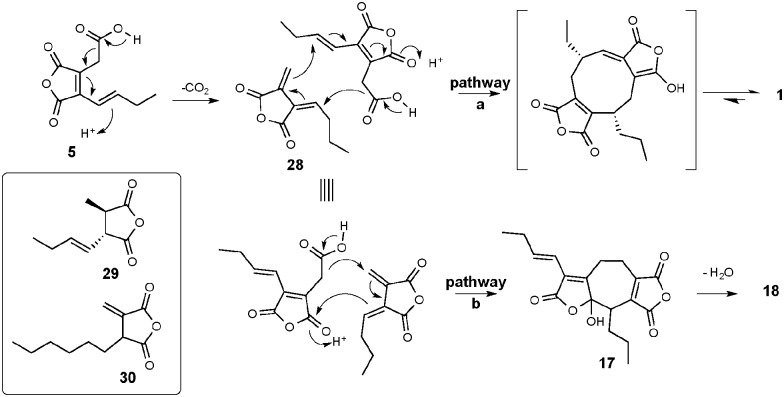
Proposed biosynthesis of nonadride and heptadrides *via* common intermediates.

Our work with *B. fulva* has led to the isolation of 5 new maleic anhydride-containing compounds in addition to byssochlamic acid. These include dihydrobyssochlamic acid, but more significantly, two novel 7-membered ring analogues which we have termed heptadrides by analogy to nine-membered nonadrides and eight-membered octadrides. Heptadrides have never been reported previously as natural products.‡
‡Tropolone metabolites, *e.g.* stipitatonic and puberlonic acids, contain maleic anhydride moieties fused to 7-membered aromatic polyketide-derived rings, in contrast to the alicyclic rings found in the nonadride and related metabolites. In addition, the putative monomeric maleic anhydride intermediate previously proposed to undergo macrocyclisation *via* dimerisation to give byssochlamic acid and other nonadrides, has also been isolated for the first time. A scheme for the biosynthesis of both the nonadrides and heptadrides is proposed. Work is in progress to establish details of the biosynthesis, as well as the identification of the biosynthetic gene cluster.

To account for diversity among nonadride-like natural products, we propose the generic name *maleidrides* to denote biosynthetically related compounds, with one (monomaleidrides) or two (bismaleidrides) maleic anhydride units anchored on a diversely substituted ring. Production of the anhydride **5** by *B. fulva*, along with hepta- and nonadrides, indicates that the diversity among maleidrides very likely arises not only from diversely substituted anhydride precursors, but also from alternative dimerisation modes.

We thank Prof. Matthew Crump for help with acquiring 600 MHz NMR data, Dr Craig Butts and Paul Lawrence for assistance with 500 MHz-cryo NMR instrument and Dr Paul Gates from Bristol University Mass Spectrometry Service for help with obtaining HRMS data. Financial support (AJS and KW) was provided by BBSRC and Syngenta (BB/J006289/1), and the EPSRC Bristol Chemical Synthesis Centre for Doctoral Training, EP/G0367641/1(studentship for DEO'F).
