# Supplementary material for: Novel nonadride, heptadride and maleic acid metabolites from the byssochlamic acid producer Byssochlamys fulva IMI 40021 – an insight into the biosynthesis of maleidrides
Source: Chem Commun (Camb). 2015 Oct 9;51(96):17088–91. doi: 10.1039/c5cc06988b (PMC4766579; doi:10.1039/c5cc06988b)
Supplement: Supplementary file 1 [file CC-051-C5CC06988B-s001.pdf]

Electronic Supplementary Material (ESI) for ChemComm.
This journal is © The Royal Society of Chemistry 2015

Experimental and Supporting Information

Novel nonadride, heptadride and maleic acid metabolites from the byssochlamic acid producer *Byssochlamys fulva* IMI 40021 – an insight into the biosynthesis of maleidrides

Agnieszka J. Szwalbe[a], Katherine Williams[a], Daniel E. O'Flynn[a], Andrew M. Bailey[b], Nicholas P. Mulholland[c], Jason E. Vincent[c], Christine L. Willis[a], Russell J. Cox[*a,d] and Thomas J. Simpson[*a].

[a] School of Chemistry, Bristol University, Cantock's Close, Bristol, BS8 1TS, UK

[b] School of Biological Sciences, University of Bristol, Bristol Life Sciences Building, 24 Tyndall Avenue, Bristol,

BS8 1TQ, UK

[c] Syngenta, Jealott's Hill International Research Centre, Bracknell, Berkshire, RG42 6EY, United Kingdom

[d] Leibniz Universität Hannover, Institute of Organic Chemistry, Schneiderberg 1B, 30167 Hannover, Germany

Experimental and Supporting Information Contents

General experimental 3

Fungus 3

Production medium 3

Fermentation conditions 3

Extraction and isolation 3

Isolated metabolites 4

Table S1. ^{1}H and ^{13}C NMR (500MHz) data of **1** and **2** 5

Table S2. ^{1}H and ^{13}C NMR (500MHz) data of **17** and **18** 6

Table S3. ^{1}H and ^{13}C NMR (500MHz) data of **5** and **6** 7

Figure S1. DAD chromatograms showing decarboxylation of anhydride **5** to **6** 8

Figure S2. UV and ESIMS spectra of identified metabolites acquired during LCMS analysis 9

Figure S3. ^{1}H NMR ($CDCl_3$) spectrum of byssochlamic acid **1** 11

Figure S4. ^{13}C NMR ($CDCl_3$) spectrum of byssochlamic acid **1** 12

Figure S5. ^{1}H NMR (DMSO-d_6) spectrum of 10-dihydrobyssochlamic acid **2** 13

Figure S6. ^{13}C NMR (DMSO-d_6) spectrum of 10-dihydrobyssochlamic acid **2** 14

Figure S7. ^{1}H NMR ($CDCl_3$) spectrum of agnestadride A **17** 15

Figure S8. ^{13}C NMR ($CDCl_3$) spectrum of agnestadride A **17** 16

Figure S9. ^{1}H NMR ($CDCl_3$) spectrum of agnestadride B **18** 17

Figure S10. ^{13}C NMR ($CDCl_3$) spectrum of agnestadride B **18** 18

Figure S11. ^{1}H NMR ($CDCl_3$) spectrum of anhydride **5** 19

Figure S12. ^{13}C NMR ($CDCl_3$) spectrum of anhydride **5** 20

Figure S13. ^{1}H NMR ($CDCl_3$) spectrum of anhydride **6** 21

Figure S14. ^{13}C NMR ($CDCl_3$) spectrum of anhydride **6** 22

Synthetic procedures 23

Figures S15-S28. ^{1}H and ^{13}C NMR spectra of synthetic intermediates 29

General experimental LCMS with autopurification system comprising Waters 2767 autosampler, Waters 515 HPLC pump, Waters 2998 Diode Array detector, Waters 2424 ELS detector and Waters Quatro Micro mass spectrometer, equipped with guard pre-column. Analytical column: Phenomenex, Kinetex, 5 μ, C18, 100Å, 250 x 4.60 mm; flow rate 1 ml/min. Preparative column: Phenomenex, Kinetex, 5 μ, C18, 100 Å, 250 x 21.20 mm; flow rate 16 ml/min; gradient programmes (30 min) :(i) **Figure 1**: 40-90%; 0 min. - 5% ACN, 2 min. - 40% ACN, 20 min. - 90% ACN, 22 min. - 95% ACN, 26-30 min. – 5% ACN; (ii) **Figure** S1: 15-60%; 0 min. - 5% ACN, 2 min. - 5% ACN, 20 min. - 60% ACN, 22 min. - 95% ACN, 26-30 min. – 5% ACN. All chromatographic solvents used were of HPLC grade and contained 0.05% of formic acid.

NMR instruments: Varian 400-MR (400MHz), Varian VNMRS500 (500MHz), Bruker 500 Cryo (500MHz) or Varian VNMRS600 Cryo (600MHz).

HRESIMS data was obtained on: [a]Bruker Daltonics micrOTOF II, [b]Bruker Daltonics Apex IV FT-ICR instruments.

Optical rotations were recorded using the sodium D line (λ = 589 nm) on a Bellingham and Stanley ADP220 polarimeter.

Producing organism *Byssochlamys fulva* strain Olliver & G. Sm. (IMI 40021) from the CABI culture collection.

Production medium Czapex Dox Broth medium, 5% (v/v) of solution A (sodium nitrate 40g/l, potassium chloride 10g/l, magnesium sulphate heptahydrate 10g/l, ferrous sulphate heptahydrate), 5% (v/v) of solution B (di-potassium hydrogen ortophosphat 20g/l), 0.1% (v/v) of solution C (zinc sulphate heptahydrate), 0.1% (v/v) of solution D (cupric suplhate pentahydrate 0.5g) and 3% (w/v) of D-glucose. The medium was sterilized (120°C, 4h) prior to inoculation.

Fermentation conditions Stock of *B. fulva* was grown Potato Dextrose Agar plates (Sigma-Aldrich). For metabolite production, 500 ml Erlenmeyer flask containing 100 ml of sterile Czapek Dox medium was inoculated with a plug of agar from a stock plate, left untouched on a shelf in a constant temperature room at 25ºC for 20-50 days of fermentation before extraction.

Extraction and isolation Liquid culture from multiple flasks was combined and mycelia separated at reduced pressure on a Büchner funnel equipped with a cellulose filter. The filtered culture liquid was then acidified with 35% HCl (0.5 ml per 100 ml of liquid) and extracted twice with ethyl acetate. Organic fractions were combined and concentrated on a rotary evaporator (water bath temperature: 30^{0}C) to yield a crude extract, which was re-dissolved in HPLC-grade MeCN prior to LCMS analysis. All compounds were isolated by preparative LCMS system.

Isolated metabolites

Byssochlamic acid **1 -** white solid; $[\alpha]^{19}$ = +91.7^0 (2.4 mg·ml^{-1}, $CHCl_3$), (lit. $[\alpha]^{23}$ = +101^0, 2.4 mg·ml^{-1}, $CHCl_3$),[i] ; t_R 15.4 min; λ_{max} (LCMS) 210, 250 nm; negative ESIMS(LCMS) *m/z* 331.6 [M-H]$^-$; ^{1}H and ^{13}C NMR data see: Table S1.

10-dihydrobyssochlamic acid **2 -** isolated as whitish solid; $[\alpha]^{20}$ = +45.7^0 (5.0 mg·ml^{-1} in acetone);; t_R 11.2 min; λ_{max} (LCMS) 211, 260 nm; negative ESIMS (LCMS) *m/z* 333.6 [M-H], 667.9 [2M-H]$^-$; negative HRESIMS[b] *m/z* 333.1344 [M-H]$^-$ ($C_{18}H_{21}O_6$ requires 333.1338); ^{1}H and ^{13}C NMR data see: Table S1.

Agnestadride A **17 -** whitish solid; t_R 13.2 min; λ_{max} (LCMS) 215, 258 nm; negative ESIMS (LCMS) *m/z* 165.6*,* 331.6 [M-H]$^-$, 663.9 [2M-H]$^-$; negative HRESIMS[a] *m/z* 331.1180 [M-H]$^-$ ($C_{18}H_{19}O_6$ requires 331.1182); ^{1}H and ^{13}C NMR data see: Table S2.

Agnestadride B **18 -** bright yellow solid, t_R 18.4 min; λ_{max} (LCMS) 218, 257, 311 nm; negative ESIMS (LCMS) *m/z* 313.6 [M-H]$^-$, 331.7 [M-H+H_2O]; positive HRESIMS[a] *m/z* 337.1053 [M+Na]$^+$ ($C_{18}H_{18}NaO_5$ requires 337.1052); ^{1}H and ^{13}C NMR data see: Table 2.

Anhydride **5 -** yellowish oil; t_R 7.6 min; λ_{max} (LCMS) 313 nm; negative ESIMS (LCMS) *m/z* 165.5 [M-H-CO_2]$^-$, 209.5 [M-H]$^-$; positive ESIMS (LCMS) *m/z* 211.5 [M+H]$^+$, 193.3 [M+H-H_2O]$^+$; ^{1}H and ^{13}C NMR data see: Table S3.

Anhydride **6 -** yellowish oil; t_R 14.7 min; λ_{max} (LCMS) 313 nm; negative ESIMS (LCMS) *m/z* 165.5 [M H]$^-$; positive HRESIMS[a] *m/z* 189.0522 [M+Na]$^+$ ($C_9H_{10}NaO_3$ requires 189.0528); ^{1}H and ^{13}C NMR data see: Table S3.

[i] J. D. White, K, Jungchul, N. E. Drapela, J. Am. Chem. Soc., 2000, 122, 8665.

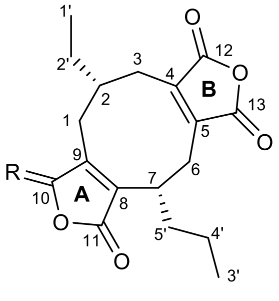

1 R = O
2 R = H,OH

	Byssochlamic acid **1**			10-dihydrobyssochlamic acid **2**		
Position	δ_H (*J* in Hz)	δ_C (J in Hz)	HMBC	δ_H (J in Hz)	δ_C (J in Hz)	HMBC
1	2.29 d, 2.72 m	30.2	2,3,2’,10,8,9	2.42, 2.32	31.2	2,3,8,9,10,2’
2	1.83-1.98 bs	40.4		1.38	37.2	1’, 2’
3	2.63 m, 2.35 m	29.5	1,5	2.89, 2.18	39.3	12,4,5,2,2’
4	-	144.3	-	-	139.1	-
5	-	143.3	-	-	140.9*	-
6	2.85, 2.90 o/m	28.2	4,5,7,13	3.03, 2.60	37.8	13,8,4,5’,5,7
7	3.40 m	34.9	6, 8,9,11	2.81 m	33.8	5’,8,9, 11
8	-	144.7	-	-	130.1	-
9	-	143.6	-	-	160.3	-
10	-	165.6	-	5.71	95.9	11, 8
11	-	165.4	-	-	171.8	-
12	-	165.4	-	-	169.3	-
13	-	165.0	-	-	168.5	-
1’	1.12 t (7.3)	11.7	2’, 2	0.92 t (7.2)	11.3	2,2’
2’	1.64 m, 1.55 m	30.2	1’,2,3,1	1.35	30.8	1’,3,2
3’	0.95 t (7.3)	13.9	4’, 5’	0.87 t (7.3)	13.9	3’, 5’
4’	1.43 m, 1.35 m	20.8	3’, 7	1.25 m	20.5	3’,5’,7

5’	1.69 m	36.3	8,3’,4’,6,7	1.50, 1.46 o/m	37.0	4’,6,7,8

Solvents: [a]$CDCl_3$, [b]dmso-d_6; *o/m* – overlapping multiplets, *bs* – broad singlet; * visible in HMBC

Table S1. ^{1}H (500 MHz) and ^{13}C NMR (125 MHz) data of **1** and **2**.

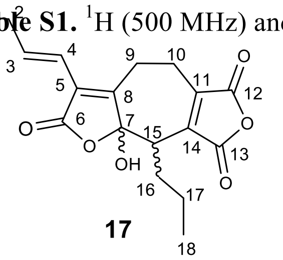

17

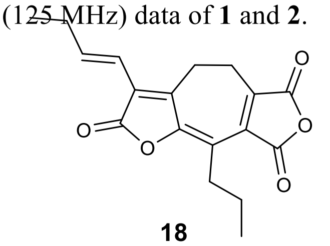

18

	Agnestadride A **17**			Agnestadride B **18**		
Position	δ_H (*J* in Hz)[a]	δ_C (J in Hz)[b]	HMBC[b]	δ_H (*J* in Hz)[c]	δ_C (J in Hz)[c]	HMBC[c]
1	1.10 (t, 7.5)	13.0	2,3	1.11 (t, 7.4)	13.0	3, 2
2	2.26 (dq)	27.1	1,3,4,5,8	2.30 (dq)	27.5	1,4, 3
3	7.06 (dt, 15.85, 6.6)	143.5	1,2,5	7.12 (dt, 15.86, 6.76)	145.7	1, 2, 5
4	6.08 (dt, 15.85, 1.6)	115.3	2,3,5,6,8	6.18 (dt, 15.7, 1.67)	116.5	5, 2,3,6
5	-	127.0	-	-	126.42	
6	-	168.55	-	-	166.2	
7	-	104.0	-	-	140.0	
8	-	153.4	-	-	142.2	
9	3.01, 2.66 (m)	22.9	5,7,8,10,11	1.25, 2.91	29.8 20.9	
10	3.05, 2.52 (m)	20.6	8,9,11,12, 14	2.85		
11	-	143.3	-	-	138.3	
12	-	165.3	-	-	164.6	
13	-	164.8	-	-	163.1	
14	-	144.9	-	-	155.8	
15	3.54 (dd, 10.3, 4.1)	43.8	7,8,11,13, 14,16,17	-	118.7	
16	1.54, 1.22 (m)	31.0	7,14,15,17,18	2.86	29.8	

17	1.31, 1.19 (m)	20.8	15,16,18	1.51, 2.86	23.4	16, 18, 15
18	0.88 (t, 7.24)	14.0	17, 16	0.99 (t, 7.4)	14.1	17, 16

Instruments: [a]500MHz, [b]600MHz, [c]500MHz-cryo

	Anhydride **5**		Anhydride **6**	
Position	δ_H (*J* in Hz)	δ_C (J in Hz)	δ_H (*J* in Hz)	δ_C (J in Hz)
1	1.12 (t, 7.4)	12.5	1.12 (t, 7.4)	12.5
2	2.34 (dq, 6.9, 1.6)	27.7	2.32 (dq)	27.6
3	7.32 (dt, 15.8, 6.6)	152.0	7.17 (dt, 15.9, 6.8)	149.1
4	6.24 (dt, 15.8, 1.67)	116.0	6.23 (dt, 16.0, 1.7)	116.2
5	-	139.9	-	137.7

Table S2. ^{1}H and ^{13}C NMR data of **17** and **18** ($CDCl_3$).

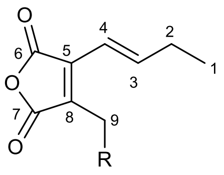

5 R = CO_2 H
6 R = H

6	-	164.0	-	164.9
7	-	165.5	-	166.6
8	-	129.6	-	135.4
9	3.61 (s)	29.6	2.11 (s)	9.3
10	-	172.2	-	-

-

Table S3. ^{1}H (400 MHz) and ^{13}C NMR (100 MHz) data of **5** and **6** ($CDCl_3$).

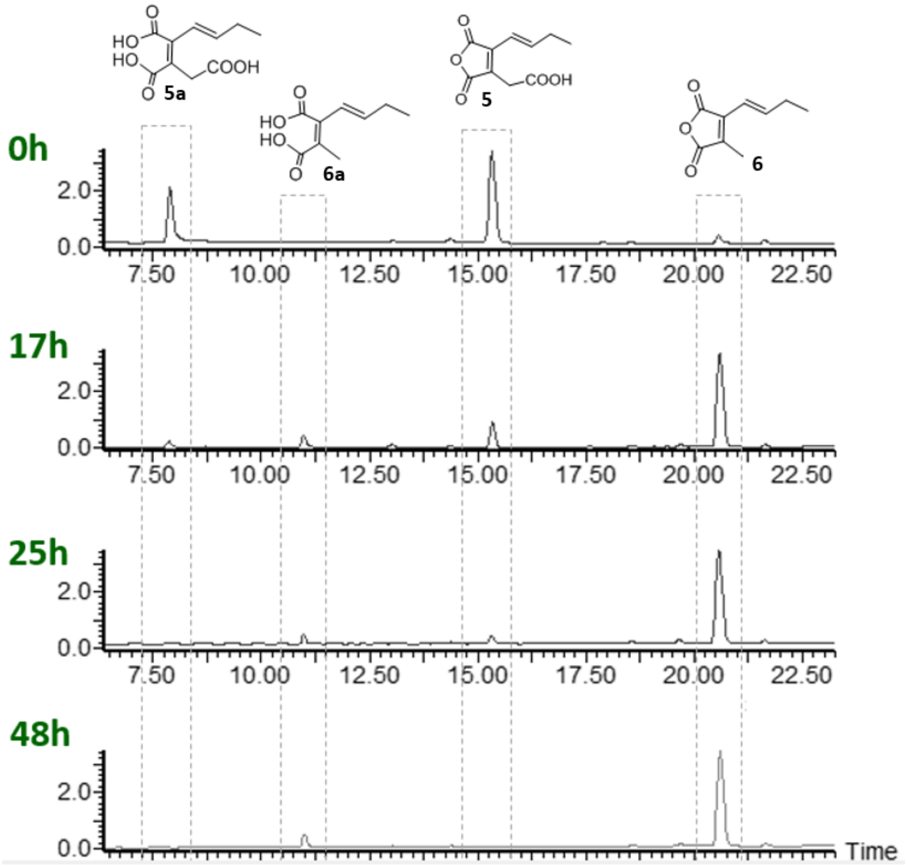

Figure S1. DAD chromatograms showing decarboxylation of anhydride **5** to **6** occurring with time.
